# Supplementary material for: Omicron sub-lineage BA.5 infection results in attenuated pathology in hACE2 transgenic mice
Source: Commun Biol. 2023 Sep 13;6:935. doi: 10.1038/s42003-023-05263-6 (PMC10499788; doi:10.1038/s42003-023-05263-6)
Supplement: Supplementary file 2 — Supplementary Information [file 42003_2023_5263_MOESM2_ESM.pdf]

Supplement figure legends

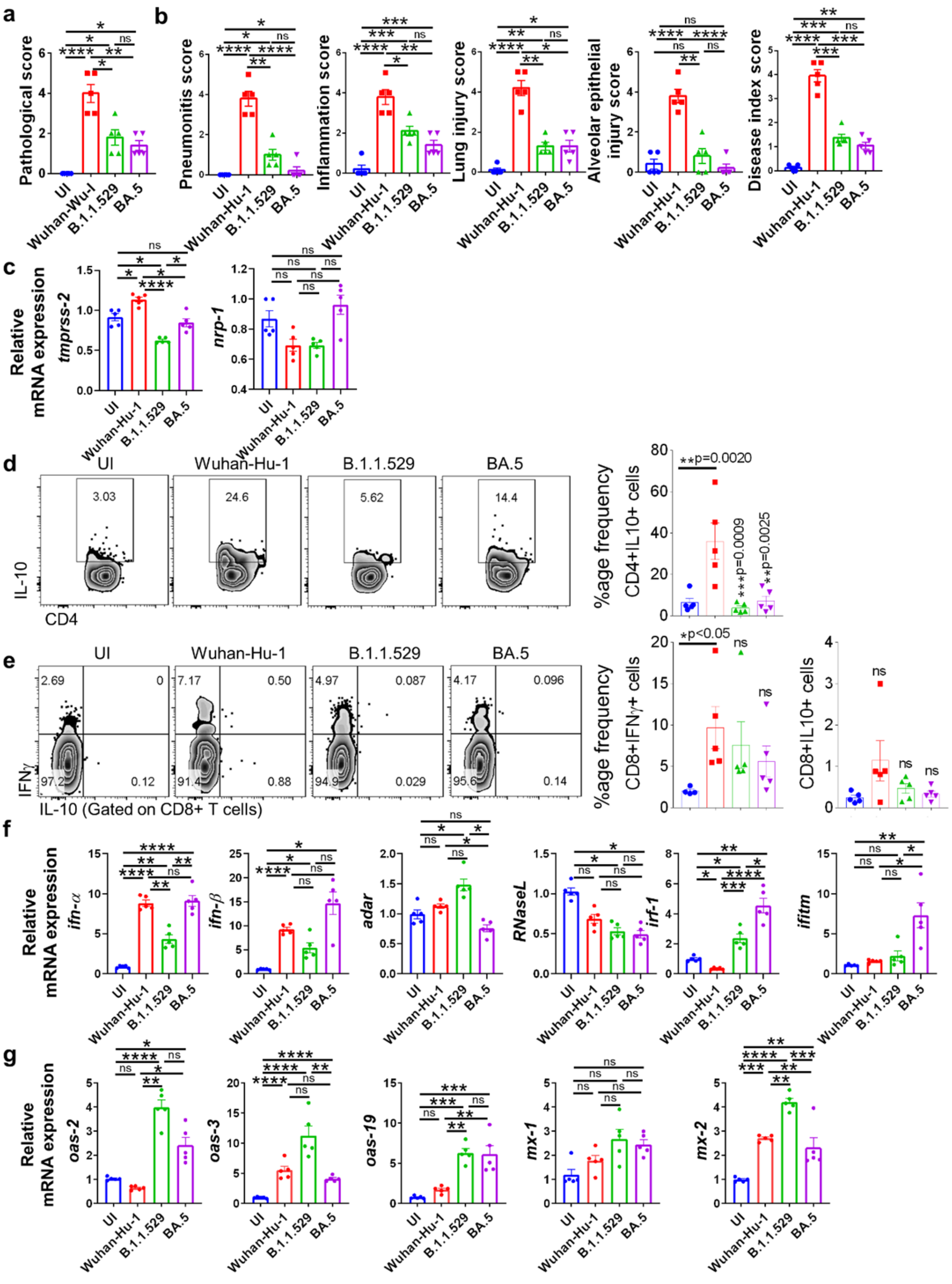

Supplement Fig. 1. Characterization of immunopathology of BA.5 infection hACE2.Tg

mice. Random blind assessment of the HE stained lung sections were done by a trained pathologist for different challenge groups and were given pathological scores based on a scale 0 to 5 (where 0 meant no feature and 5 meant highest pathological feature). (a) Bar graph showing mean $\pm$ SEM score for lung pathological features and disease index score calculated as the average of all features for that animal. (b) mRNA expression of nrp-1 & temprss-2 genes from the lung samples. (c) intracellular cytokines levels of IL-10 producing CD4+ T cells (d) or IFNγ/ IL-10 producing CD8+ T cells in the BALF. (e) Relative mRNA expression of (f) interferon stimulating genes (ISGs) and (g) anti-viral genes in the lung samples of infected or uninfected mice. For all experiment n=5. One way-Anova using non-parametric Kruskal-Wallis test for multiple comparison. ns= non-significant, *P < 0.05, **P < 0.01, ***P < 0.001, ****P < 0.0001.

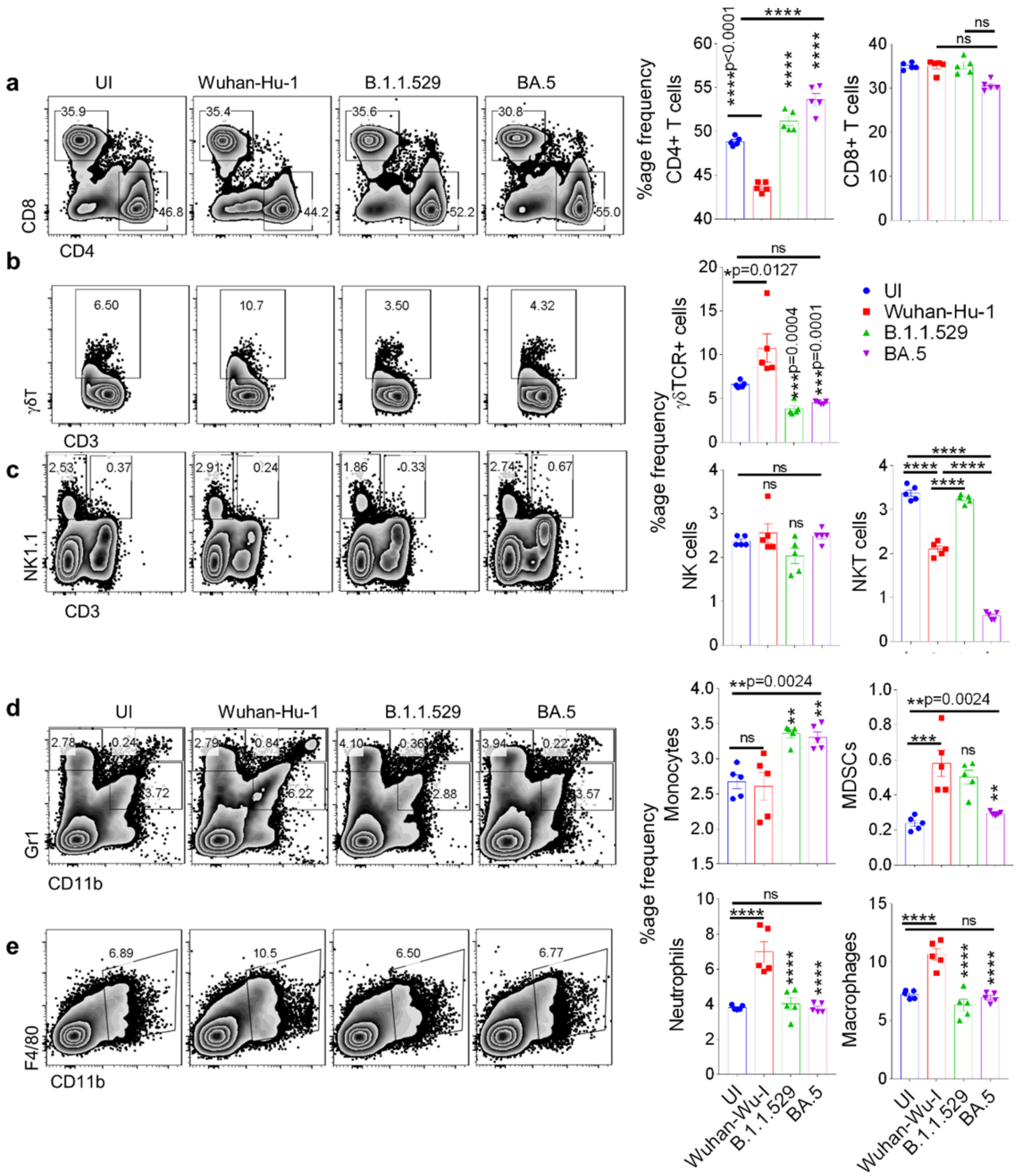

Supplement Fig. 2. Evaluation of the immunological response in BA.5 infected mice. Splenocytes of the infected or uninfected animals were used for the (a-e) immunophenotyping by flow cytometry to investigate the changes in the percent frequency of (a) CD4+ or CD8+ T cells (b) γδTCR+ T cells (c) NK and NKT cells (d) monocytes, MDSCs and neutrophils and (e) macrophages shown through representative dot plots and bar graphs. For all experiment

n=5. One way-Anova using non-parametric Kruskal-Wallis test for multiple comparison. ns= non-significant, *P < 0.05, **P < 0.01, ***P < 0.001, ****P < 0.0001.

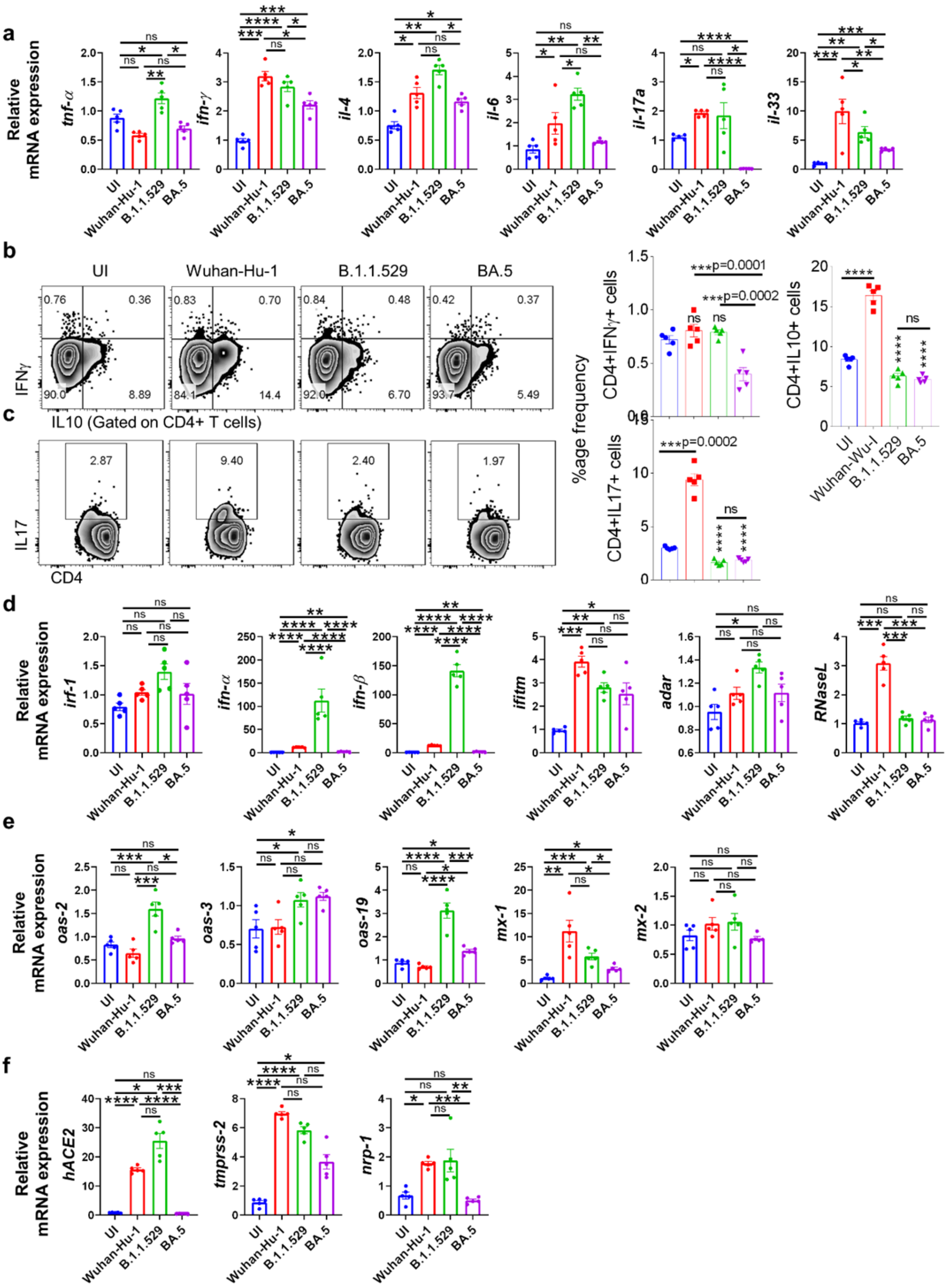

Supplement Fig. 3. Inflammatory and anti-viral response in BA.5 infected mice Splenocytes of the infected vs uninfected mice were used for relative mRNA expression profiling or flow cytometry to evaluate the inflammatory and anti-viral response following BA.5 infection in hACE2 transgenic mice. (a) mRNA expression of pro-inflammatory cytokines genes (b) representative dot plots and bar graphs showing mean $\pm$ SEM of percentage frequency of IFNγ, IL-10 or (c) IL17A producing CD4+ T cells (d) relative mRNA expression of ISGs genes and (e) anti-viral genes or (f) SARS-CoV-2 host entry factors. For all experiment n=5. One way-Anova using non-parametric Kruskal-Wallis test for multiple comparison. ns= non-significant, *P < 0.05, **P < 0.01, ***P < 0.001, ****P < 0.0001.

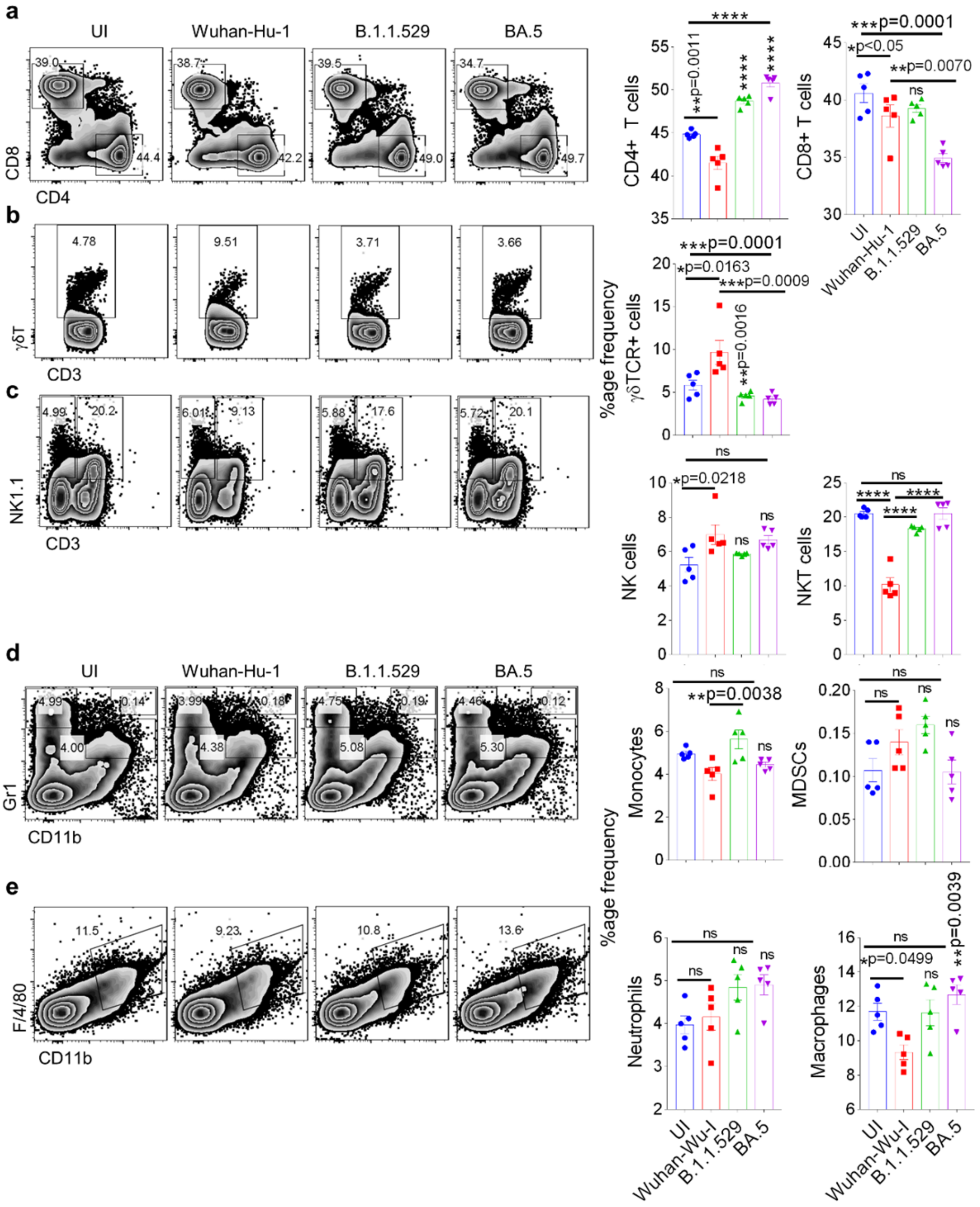

a
UI
Wuhan-Hu-1
B.1.1.529
BA.5
CD8
CD4
CD4+ T cells
CD8+ T cells
b
γδT
CD3
%age frequency γδTCR+ cells
c
NK1.1
CD3
NK cells
NKT cells
d
Gr1
CD11b
Monocytes
MDSCs
e
F/4/80
CD11b
%age frequency
Neutrophils
Macrophages

33

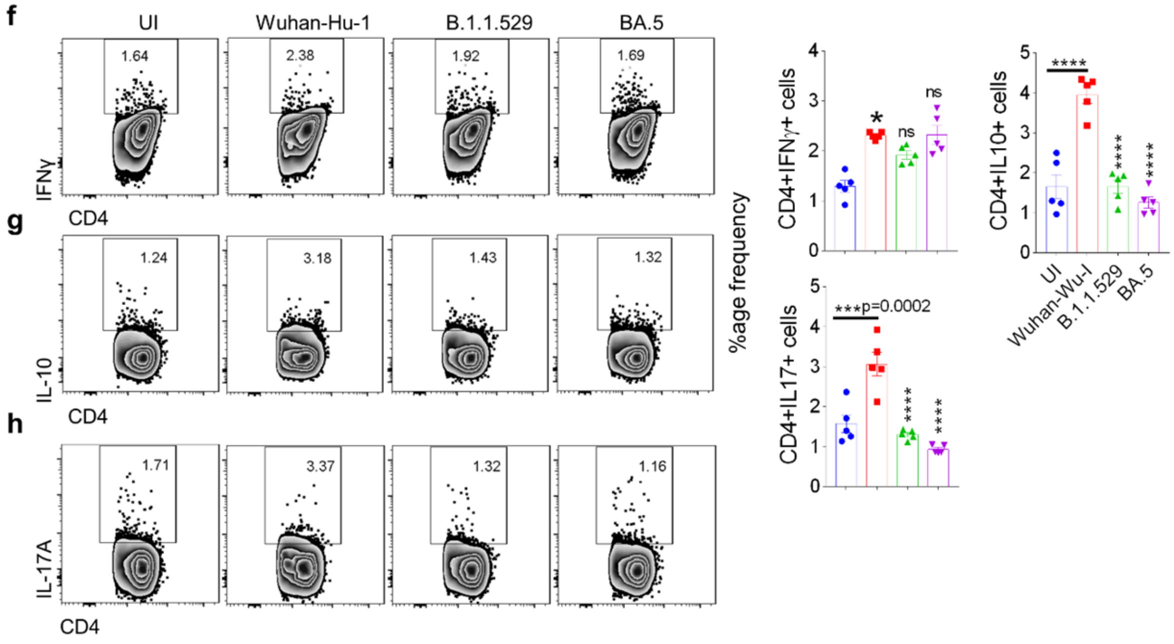

Supplement Fig. 4. Characterization of the immunological response in the dLN of BA.5 infected mice. Lymph nodes of the infected or uninfected animals were used for the immunophenotyping assessment by flow cytometry to investigate the changes in the percent frequency of (a) CD4+ or CD8+ T cells (b) γδTCR+ T cells (c) NK and NKT cells (d) monocytes, MDSCs and neutrophils and (e) macrophages (f) IFNγ, (g) IL-10 or (h) IL17A producing CD4+ T cells. For all experiment n=5. One way-Anova using non-parametric Kruskal-Wallis test for multiple comparison. ns= non-significant, *P < 0.05, **P < 0.01, ***P < 0.001, ****P < 0.0001.

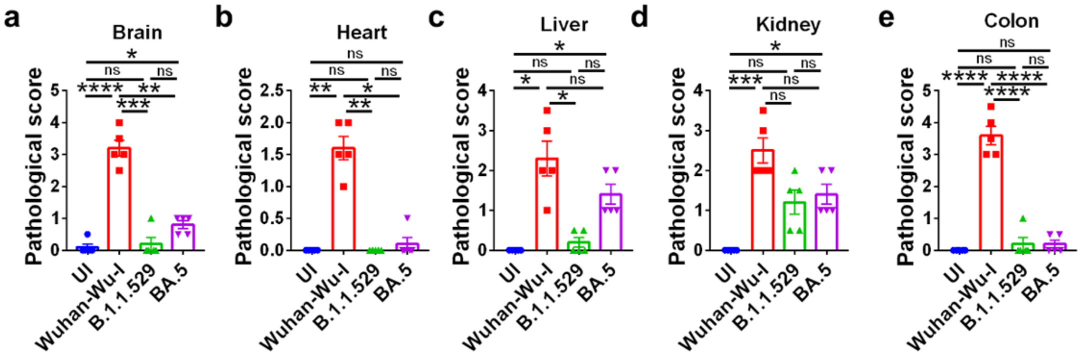

Supplement Fig. 5. Pathological scores for extra-pulmonary manifestations induced by

BA.5 infection. Random blind assessment of the HE stained sections of major organs: (a) brain (b) heart (c) liver (d) kidney (e) colon was done by a trained pathologist for different challenge groups and were given cumulative pathological scores which included inflammation, based on a scale 0 to 5 (where 0 meant no feature and 5 meant highest pathological feature). For all experiment $n=5$. One way-Anova using non-parametric Kruskal-Wallis test for multiple comparison. ns= non-significant, *$P < 0.05$, **$P < 0.01$, ***$P < 0.001$, ****$P < 0.0001$.

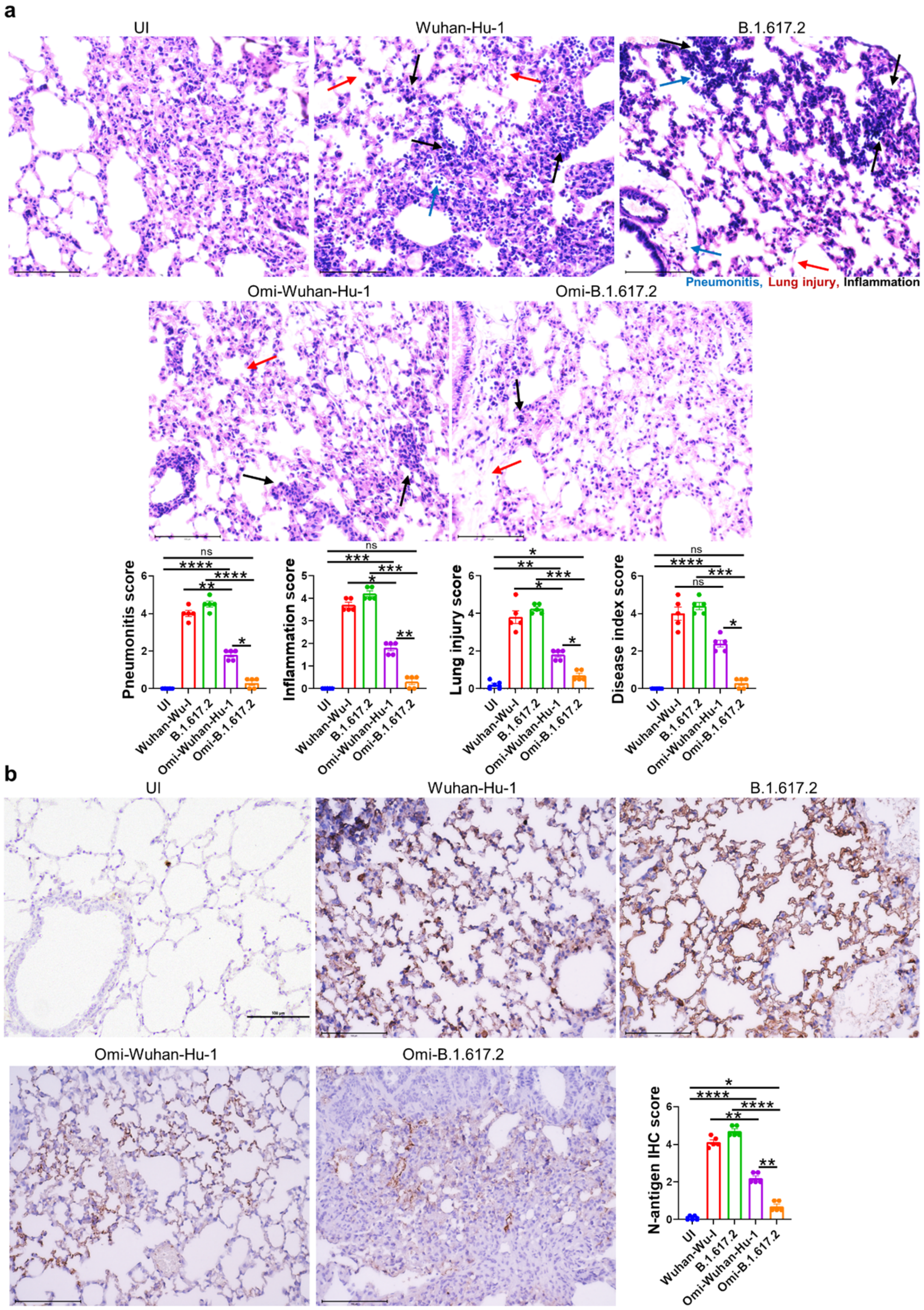
a
UI
Wuhan-Hu-1
B.1.617.2
Pneumonitis, Lung injury, Inflammation
Omi-Wuhan-Hu-1
Omi-B.1.617.2
Pneumonitis score
Inflammation score
Lung injury score
Disease index score
ns
****
**
*
***
UI
Wuhan-Wu-I
B.1.617.2
Omi-Wuhan-Hu-1
Omi-B.1.617.2
b
UI
Wuhan-Hu-1
B.1.617.2
Omi-Wuhan-Hu-1
Omi-B.1.617.2
N-antigen IHC score

c

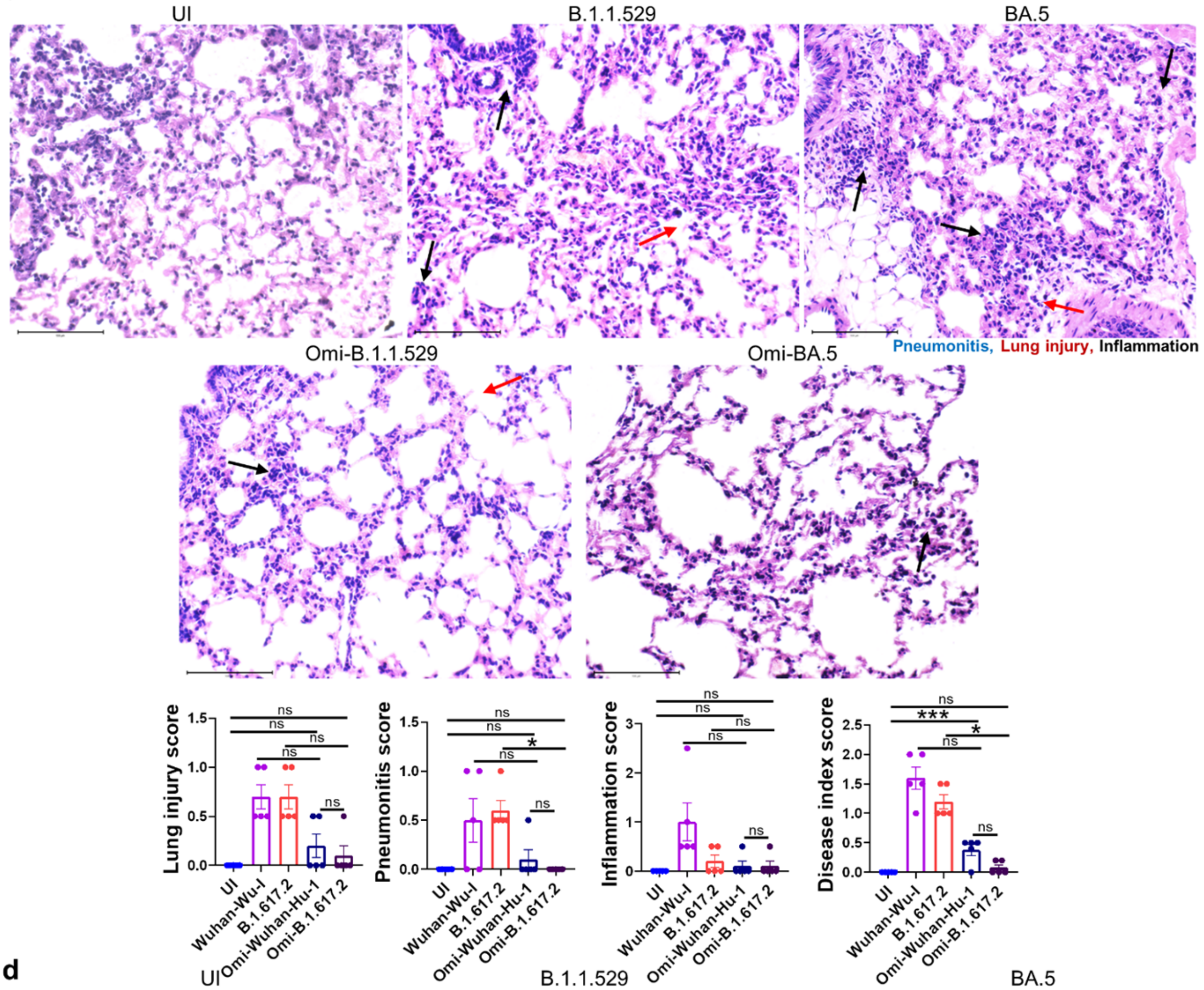
UI
B.1.1.529
BA.5
Pneumonitis, Lung injury, Inflammation
Omi-B.1.1.529
Omi-BA.5
Lung injury score
Pneumonitis score
Inflammation score
Disease index score
ns
*
***
UI
Wuhan-Wu-I
B.1.617.2
Omi-Wuhan-Hu-1
Omi-B.1.617.2

d

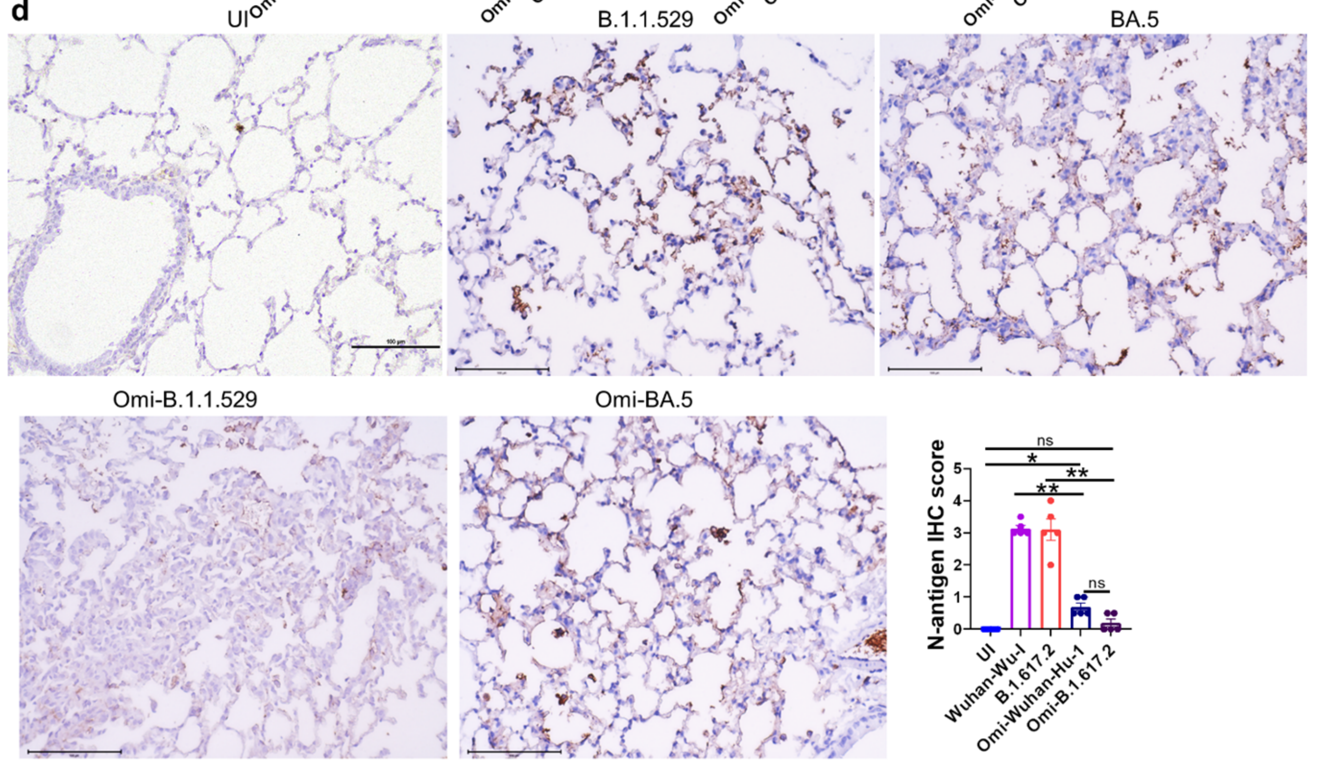
UI
B.1.1.529
BA.5
Omi-B.1.1.529
Omi-BA.5
N-antigen IHC score
ns
*
**
UI
Wuhan-Wu-I
B.1.617.2
Omi-Wuhan-Hu-1
Omi-B.1.617.2

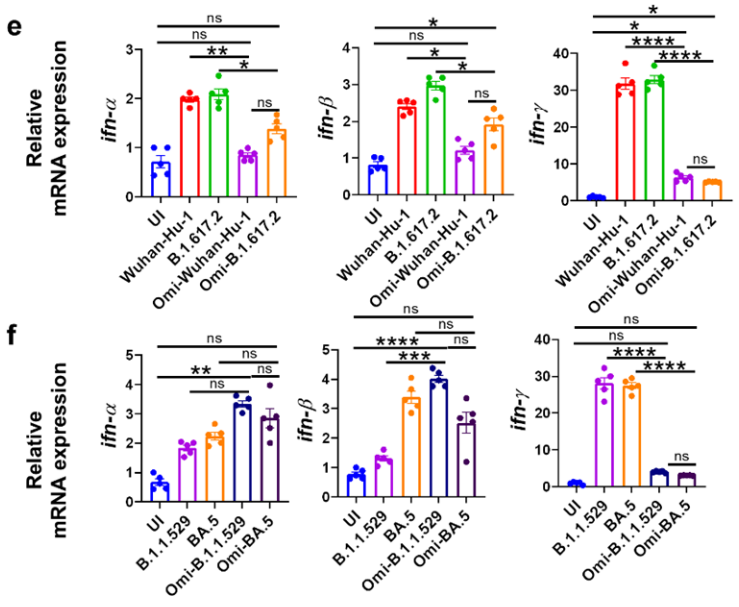

Supplement Fig. 6. Histopathological changes and inflammatory cytokine mRNA expression in the lungs of re-infected mice. 14 mice post omicron infection was used as recovery model for the re-infection studies with different VoCs: (a & b) Wuhan reinfection (Omi-Wuhan-Hu-1), B.1.617.2 reinfection (Omi-B.1.617.2), (c & d) B.1.1.529 reinfection (Omi-B.1.1.529) and BA.5 reinfection (Omi-BA.5). (a & c) Representative H & E stained images of lung section at 40X showing pneumonitis (blue arrow), lung injury (red arrow), inflammation (black arrow). Random blind assessment of the HE stained lung sections were done by a trained pathologist for different challenge groups and were given pathological scores based on a scale 0 to 5 (where 0 meant no feature and 5 meant highest pathological feature). (b & d) Representative IHC images for N antigen (black arrow) in the lung sections. Blinded scoring of IHC stain by trained pathologist on the scale of 0 to 5 (where 0 meant no stain & 5 meant highest brown color stain distribution). RNA from the lungs samples from rechallenged mice recovered from Omicron infection was used to evaluate the mRNA expression of IFNγ, IFNα and IFNβ genes in (e) Omi-Wuhan-Hu-1 & Omi-B.1.617.2 mice (f) Omi-B.1.1.529 & Omi-BA.5 groups.For all experiment n=5. One way-Anova using non-parametric Kruskal-

Wallis test for multiple comparison. ns= non-significant, *P < 0.05, **P < 0.01, ***P < 0.001, ****P < 0.0001.
