# Supplementary material for: Omicron sub-lineage BA.5 infection results in attenuated pathology in hACE2 transgenic mice
Source: Commun Biol. 2023 Sep 13;6:935. doi: 10.1038/s42003-023-05263-6 (PMC10499788; doi:10.1038/s42003-023-05263-6)
Supplement: Supplementary file 3 — Reporting Summary [file 42003_2023_5263_MOESM3_ESM.pdf]

nature portfolio

Corresponding author(s): Amit Awasthi, Zaigham Abbas Rizvi

Last updated by author(s): May 24, 2023

Reporting Summary

Nature Portfolio wishes to improve the reproducibility of the work that we publish. This form provides structure for consistency and transparency in reporting. For further information on Nature Portfolio policies, see our Editorial Policies and the Editorial Policy Checklist.

Statistics

For all statistical analyses, confirm that the following items are present in the figure legend, table legend, main text, or Methods section.

n/a	Confirmed	
☐	☒	The exact sample size (*n*) for each experimental group/condition, given as a discrete number and unit of measurement
☐	☒	A statement on whether measurements were taken from distinct samples or whether the same sample was measured repeatedly
☐	☒	The statistical test(s) used AND whether they are one- or two-sided<br>*Only common tests should be described solely by name; describe more complex techniques in the Methods section.*
☐	☒	A description of all covariates tested
☐	☒	A description of any assumptions or corrections, such as tests of normality and adjustment for multiple comparisons
☐	☒	A full description of the statistical parameters including central tendency (e.g. means) or other basic estimates (e.g. regression coefficient) AND variation (e.g. standard deviation) or associated estimates of uncertainty (e.g. confidence intervals)
☐	☒	For null hypothesis testing, the test statistic (e.g. *F*, *t*, *r*) with confidence intervals, effect sizes, degrees of freedom and *P* value noted<br>*Give P values as exact values whenever suitable.*
☒	☐	For Bayesian analysis, information on the choice of priors and Markov chain Monte Carlo settings
☒	☐	For hierarchical and complex designs, identification of the appropriate level for tests and full reporting of outcomes
☒	☐	Estimates of effect sizes (e.g. Cohen's *d*, Pearson's *r*), indicating how they were calculated

Our web collection on statistics for biologists contains articles on many of the points above.

Software and code

Policy information about availability of computer code

Data collection	1. Flow cytometry data were collected on BD FACSCanto II with FACSDiva software version 8.0.2 (BD).<br>2. qRT-PCR data were collected on Fast 7500 Dx qPCR system (Applied Biosystems) and analyzed using SDS 2.1 software.<br>3. ELISA data were collected at 600nm in spectrophotometer (BioLinkk).
Data analysis	1- Flow cytometry data were analyzed on FlowJo version 10(BD).<br>2. All qPCR data was analyzed using SDS 2.1 software and microsoft excel.<br>3. All the statistical analysis was performed on Prism 8 (Graph pad).<br>4. Microsoft office 2016 was used for making MS word, excel and ppt files.

For manuscripts utilizing custom algorithms or software that are central to the research but not yet described in published literature, software must be made available to editors and reviewers. We strongly encourage code deposition in a community repository (e.g. GitHub). See the Nature Portfolio guidelines for submitting code & software for further information.

Data

Policy information about availability of data

All manuscripts must include a data availability statement. This statement should provide the following information, where applicable:

- Accession codes, unique identifiers, or web links for publicly available datasets
- A description of any restrictions on data availability
- For clinical datasets or third party data, please ensure that the statement adheres to our policy

Data Availability Statement: All Data needed to evaluate the conclusions in the paper are present in the paper or the Supplementary Materials. The authors declare that all other data supporting the findings of this study are available within the article or its supplementary information files.

Research involving human participants, their data, or biological material

Policy information about studies with human participants or human data. See also policy information about sex, gender (identity/presentation), and sexual orientation and race, ethnicity and racism.

Reporting on sex and gender	NA
Reporting on race, ethnicity, or other socially relevant groupings	NA
Population characteristics	NA
Recruitment	NA
Ethics oversight	NA

Note that full information on the approval of the study protocol must also be provided in the manuscript.

Field-specific reporting

Please select the one below that is the best fit for your research. If you are not sure, read the appropriate sections before making your selection.

☒ Life sciences ☐ Behavioural & social sciences ☐ Ecological, evolutionary & environmental sciences

For a reference copy of the document with all sections, see nature.com/documents/nr-reporting-summary-flat.pdf

Life sciences study design

All studies must disclose on these points even when the disclosure is negative.

Sample size	The sample size for in vivo studies were determined based on our preliminary data. The sample size was selected to produce statistically relevant biological difference in the study. Sample sizes were determined in accordance with the literature and based on previous experience in our group.
Data exclusions	No data were excluded from the analyses.
Replication	All the experiments were replicated at least 3 times independently. For all in vivo studies biological replicates were taken.
Randomization	Randomizations of the mice were done based on their body weight or genotypes. For experiments involving genetically modified animals, litter mates were used for each experiment.
Blinding	To evaluate unbiased disease phenotype blinding was done by 3 unbiased observers. Histological score was given by professional histologist for n=5 samples. Other data presented did not require the use of blinding. Data reported for mouse experiments were not subjective but rather based on quantitative analyses. In the cell and animal experiments, investigators were not blinded to group allocation because the investigators should give the drug to the mice and cell in different treatment conditions. For flowcytometry experiments, the samples were acquired blindly.

Reporting for specific materials, systems and methods

We require information from authors about some types of materials, experimental systems and methods used in many studies. Here, indicate whether each material, system or method listed is relevant to your study. If you are not sure if a list item applies to your research, read the appropriate section before selecting a response.

Materials & experimental systems

n/a	Involved in the study
☐	☒ Antibodies
☐	☒ Eukaryotic cell lines
☒	☐ Palaeontology and archaeology
☐	☒ Animals and other organisms
☒	☐ Clinical data
☒	☐ Dual use research of concern
☒	☐ Plants

Methods

n/a	Involved in the study
☐	☐ ChIP-seq
☐	☒ Flow cytometry
☐	☐ MRI-based neuroimaging

Antibodies

Antibodies used

Anti-mouse monoclonal antibodies were used for flow cytometry accessement: α-CD3-BV510 (145-2C11, Biolegend, 1:600), α-CD4-PerCp-Cy5.5 (GK1.5, Biolegend, 1:1000), α-CD8-BV410 (53-6.7, Biolegend, 1:1000), α-γδTCR FITC (UC7-13D5, Biolegend, 1:500), α-NK1.1 APC (S17016D, Biolegend, 1:700), α-IFN-γ-PE (XMG1.2, Biolegend, 1:400), IL17A-APC (TC11-18H10, Biolegend, 1:400), α-IL-2-PE-Cy7 (11B11, Biolegend, 1:400), IL-10-FITC (JES5-16E3, Biolegend, 1:400), α-Gr1-BV421 (RB6-8C5, Biolegend, 1:1500), α-CD11b PerCp-Cy5.5 (#101228, Biolegend, 1:1500), α-c-kit BV510 (2B8, Biolegend, 1:800), α-FcƐr1α-APC (MAR-1, Biolegend, 1:500).

Validation

All antibodies were validated by the supplier (Bio Legend, Peprotech) and were checked in the lab by comparing manufacturers or in house results. Statement from Bio legend: Bio legend Antibodies under go an extensive series of testing to ensure quality at every step in the manufacturing process, as well as maintaining quality after the sale.
Statement from Peprotech: Peprotechs monoclonal antibodies are raised against full length recombinant antigens and have been thoroughly screened for performance in variety of applications.
The validation statement and the relevant citation information is listed in the link:
1. anti-mouse CD3 BV510 (Cat no- 100353,Clone-145-2C11,Biolegend INC, USA, 2:1000) https://www.biolegend.com/en-ie/products/brilliant-violet-510-anti-mouse-cd3epsilon-antibody-11973
2. anti-mouse γδTCR FITC (Cat no-118106,Clone-GL3, , Biolegend INC,USA, 2:1000) https://www.biolegend.com/en-us/products/fitc-anti-mouse-tcr-gamma-delta-antibody-2420?GroupID=BLG3687
3. anti-mouse Gr1 BV421 (Cat no-108445,Clone-RB6-8C5, Biolegend INC, USA, 2:1000) https://www.biolegend.com/en-us/products/brilliant-violet-421-anti-mouse-ly-6g-ly-6c-gr-1-antibody-7201?GroupID=BLG4876
4. anti-mouse CD11b PerCp-Cy5.5 (Cat no-101228, Biolegend INC, USA, 1:1000) https://www.biolegend.com/en-us/products/percp-cyanine5-5-anti-mouse-human-cd11b-antibody-4257?GroupID=BLG10552
5. anti-mouse CD4-Percp cy5.5 (Cat no-100538,Clone-RM4-5, Biolegend INC, USA, 1:1000) https://www.biolegend.com/en-us/products/percp-cyanine5-5-anti-mouse-cd4-antibody-4230?GroupID=BLG4211
6. anti-mouse NK1.1–PE-Cy7 (Cat no-108714,Clone-PK136, Biolegend INC, USA, 1:1000) https://www.biolegend.com/en-us/products/pe-cyanine7-anti-mouse-nk-1-1-antibody-2840?GroupID=GROUP20
7. anti-mouse-CD8 – BV421 (Cat no-100753,Clone-53-6.7 Biolegend INC, USA,2:2000) https://www.biolegend.com/en-us/products/brilliant-violet-421-anti-mouse-cd8a-antibody-7138?GroupID=BLG6765
8. F4/80 – FITC (Cat no-123108,Clone-BM8,Biolegend INC, USA, 1:1000) https://www.biolegend.com/en-us/products/fitc-anti-mouse-f4-80-antibody-4067?GroupID=BLG5319
9. CD206 - PE(Cat no-141705,Clone-C068C2, Biolegend INC, USA,2:2000) https://www.biolegend.com/en-us/products/pe-anti-mouse-cd206-mmr-antibody-7424?GroupID=BLG9506
10. CD80 – AF647 (Cat no-305216,Clone-2D10, Biolegend INC, USA, 2:2000) https://www.biolegend.com/en-us/products/alexa-fluor-647-anti-human-cd80-antibody-3352?GroupID=BLG1908
11. CD68 – PEcy7 (#137015,Clone-FA-11, Biolegend INC, USA, 1:1000) https://www.biolegend.com/en-us/products/pe-cyanine7-anti-mouse-cd68-antibody-9124?GroupID=BLG10716
12. CD49b (#117322,Clone-N418, Biolegend INC, USA, 1:1000) https://www.biolegend.com/en-us/products/pe-anti-mouse-cd49b-antibody-299?GroupID=BLG4895
13. C-kit (#105805,Clone-2B8, Biolegend INC, USA, 1:1000) https://www.biolegend.com/en-us/products/purified-anti-mouse-cd117-c-kit-antibody-77?GroupID=BLG4276
14. Fcer1 (#134308, Clone-MAR1, Biolegend INC, USA, 1:1000) https://www.biolegend.com/en-us/products/pe-anti-mouse-fcepsilonrialpha-antibody-5950?GroupID=BLG6716
15. Siglec-f(#155528,Clone-S17007L, Biolgend INC, USA, 1:1000) https://www.biolegend.com/en-us/products/purified-anti-mouse-cd170-siglec-f-antibody-16369
16. IFNγ – AF647 (#505814,Clone-XMG1.2, Biolegend INC, USA, 1:1000) https://www.biolegend.com/fr-fr/products/alexa-fluor-647-anti-mouse-ifn-gamma-antibody-2722
17. IL-17 – PE-cy7 (#506922, Clone-TC11-18H10.1,Biolegend INC, USA, 1:1000) https://www.biolegend.com/en-us/products/pe-cyanine7-anti-mouse-il-17a-antibody-6013?GroupID=GROUP24
18. IL-10 – PE (#505008, Clone-JES5-16E3, Biolegend INC, USA, 5:1000) https://www.biolegend.com/en-us/products/pe-anti-mouse-il-10-antibody-944?GroupID=GROUP24
19. Foxp3 – AF647 (#126408,Clone-MF14, Biolegend, USA,2:1000) https://www.biolegend.com/en-us/products/alexa-fluor-647-anti-mouse-foxp3-antibody-4662
20. IL-9 – Percp-cy5.5 (#514112,Clone-RM9A4, Biolegend INC, USA, 5:1000) https://www.biolegend.com/en-us/clone-search/percp-cyanine5-5-anti-mouse-il-9-antibody-9037
21. IL-4 – PE(#504104, Clone-11B11,Biolegend, USA, 1:1000) https://www.biolegend.com/ja-jp/products/pe-anti-mouse-il-4-antibody-893?GroupID=BLG1753
22. anti-mouse CD3 FITC (# 100204, Clone-17A2, Biolegend INC, USA, 2:2000) https://www.biolegend.com/en-us/products/fitc-anti-mouse-cd3-antibody-45?GroupID=BLG6732
23. anti-mouse CD11b FITC (#101206,Clone-M1/70, Biolegend INC,USA, 1:1000) https://www.biolegend.com/en-us/products/fitc-anti-mouse-human-cd11b-antibody-347?GroupID=BLG10660

24. anti-mouse B220- FITC (#103206,Clone-RA3-6B2, Biolegend INC, USA, 2:2000) https://www.biolegend.com/en-us/products/fitc-anti-mouse-human-cd45r-b220-antibody-445?GroupID=GROUP658
25. IL-9 – APC (#514106, Clone-RM9A4, Biolegend, USA, 3:3000) https://www.biolegend.com/en-us/products/apc-anti-mouse-il-9-antibody-5980?GroupID=GROUP24

Eukaryotic cell lines

Policy information about cell lines and Sex and Gender in Research

Cell line source(s)	VeroE6 (ATCC CRL-1587), A549 (Adenocarcinomic human alveolar basal epithelial cell line; ATCC-CCL185), Caco2 cells (Colon epithelial cell; HTB-37) were kind gift from Dr. Sweety Samal.
Authentication	Authenticated by STR method
Mycoplasma contamination	All cell lines were negative for Mycoplasma contamination.
Commonly misidentified lines (See ICLAC register)	None of the used cell lines is listed in ICLAC database

Animals and other research organisms

Policy information about studies involving animals; ARRIVE guidelines recommended for reporting animal research, and Sex and Gender in Research

Laboratory animals	AB6.Cg-Tg(K18-ACE2)2Prlmn/j mice (strain:034860, Common name: K18-hACE2 mice) were procured from Jackson Laboratory and bred at THSTI. Heterozygous hACE2 transgenic mice, 6-12 weeks old and mixed gender were used for all the experiments. Laboratory animals were housed at institutional animal house Facility maintained between 19 to 26 degrees ambient temperature with 30-70% humidity and 12h light and 12h dark cycle. All animal procedures containing infection were performed in laminar flow hoods inside BSL3 facility.
Wild animals	NA
Reporting on sex	*Indicate if findings apply to only one sex; describe whether sex was considered in study design, methods used for assigning sex. Provide data disaggregated for sex where this information has been collected in the source data as appropriate; provide overall numbers in this Reporting Summary. Please state if this information has not been collected. Report sex-based analyses where performed, justify reasons for lack of sex-based analysis.*
Field-collected samples	No field collected samples were used in the study.
Ethics oversight	All the experiments were performed at infectious disease research facility (IDRF) in BSL-3 and ABSL-3 as per IBSC (Institutional Biosafety committee) guidelines. All experimental procedures involving virus challenge were approved by the Institutional Animal Ethics Committee (IAEC), IBSC and RCGM as per the guidelines of THSTI (IAEC/THSTI/217) and Department of Biotechnology, Govt. of India.

Note that full information on the approval of the study protocol must also be provided in the manuscript.

Plants

Seed stocks	NA
Novel plant genotypes	NA
Authentication	NA

ChIP-seq

Data deposition

☐ Confirm that both raw and final processed data have been deposited in a public database such as GEO.

☐ Confirm that you have deposited or provided access to graph files (e.g. BED files) for the called peaks.

Data access links *May remain private before publication.*	NA
Files in database submission	NA
Genome browser session (e.g. UCSC)	NA

Methodology

Replicates	NA
Sequencing depth	NA
Antibodies	NA
Peak calling parameters	NA
Data quality	NA
Software	NA

Flow Cytometry

Plots

Confirm that:

☒ The axis labels state the marker and fluorochrome used (e.g. CD4-FITC).

☒ The axis scales are clearly visible. Include numbers along axes only for bottom left plot of group (a 'group' is an analysis of identical markers).

☒ All plots are contour plots with outliers or pseudocolor plots.

☒ A numerical value for number of cells or percentage (with statistics) is provided.

Methodology

Sample preparation	Flow cytometry and Intracellular cytokine staining<br>SCS obtained from spleen and dLN were either surface stained by using fluorescenated anti-mouse antibodies in FACS buffer (PBS with 1% FBS) and analysed as previously described 43–45; or were in-vitro in presence of RBD (2µg/ml) or its absence (with phorbol 12-myristate13-aceate (PMA; 50 ng/ml; Sigma-Aldrich) and ionomycin (1 µg/ml; Sigma-Aldrich). RBD stimulation was performed for 6 days and were used for intracellular cytokine staining. For PMA + Ionomycin cells were activated for 4 h in presence of Monensin (#554724 Golgi-Stop, BD Biosciences). All the cells were first washed and blocked with Fc block (anti-mouse CD16/32, Biolegend) at room temperature (RT) for 20 min. Thereafter, cells were used for surface staining with α-CD3, α-CD4, α-CD8, α-CD11b, α-NK1.1, α-Gr1, α-c-kit, α-γδTCR, α-FcƐr1α for 20 min at RT in dark. Thereafter, cells were fixed in Cytofix and permeabilized with Perm/Wash Buffer using Fixation Permeabilization solution kit (#554714, BD Biosciences). Permeabilized cells were stained intracellular cytokine antibodies by using -IFNγ, α-IL17A, α-IL10 antibodies in permeabilizing buffer in dark for 20 min at RT. The acquisition of the flow cytometry data was done on (Canto II; BD Bioscience) and analysed on FlowJo software (Tree-Star).
Instrument	FACS Canto (BD Biosciences)
Software	FACS Diva software version 8.0.2 (BD), Flowjo software (10 Tree star)
Cell population abundance	Spleen (0.1 million), and dLN (0.1 million were used further for surface, intra cellular cytokine staining to identify the different cell population and cytokine levels.
Gating strategy	*Describe the gating strategy used for all relevant experiments, specifying the preliminary FSC/SSC gates of the starting cell population, indicating where boundaries between "positive" and "negative" staining cell populations are defined.*

☒ Tick this box to confirm that a figure exemplifying the gating strategy is provided in the Supplementary Information.

Magnetic resonance imaging

Experimental design

Design type	NA
Design specifications	NA
Behavioral performance measures	NA

Acquisition

Imaging type(s)	NA
Field strength	NA
Sequence & imaging parameters	NA
Area of acquisition	NA

Diffusion MRI ☐ Used ☒ Not used

Preprocessing

Preprocessing software	NA
Normalization	NA
Normalization template	NA
Noise and artifact removal	NA
Volume censoring	NA

Statistical modeling & inference

Model type and settings	NA
Effect(s) tested	NA

Specify type of analysis: ☐ Whole brain ☐ ROI-based ☐ Both

Statistic type for inference (See Eklund et al. 2016)	NA
Correction	NA

Models & analysis

n/a	Involved in the study
☒	☐ Functional and/or effective connectivity
☒	☐ Graph analysis
☒	☐ Multivariate modeling or predictive analysis
